# LC-MS-Based Metabolic Fingerprinting of Aqueous Humor

**DOI:** 10.1155/2017/6745932

**Published:** 2017-01-05

**Authors:** Karolina Pietrowska, Diana Anna Dmuchowska, Paulina Samczuk, Tomasz Kowalczyk, Pawel Krasnicki, Malgorzata Wojnar, Aleksandra Skowronska, Zofia Mariak, Adam Kretowski, Michal Ciborowski

**Affiliations:** ^1^Clinical Research Centre, Medical University of Bialystok, M. Sklodowskiej Curie 24a, 15-276 Bialystok, Poland; ^2^Department of Ophthalmology, Medical University of Bialystok, M. Sklodowskiej Curie 24a, 15-276 Bialystok, Poland

## Abstract

Aqueous humor (AH) is a transparent fluid which fills the anterior and posterior chambers of the eye. It supplies nutrients and removes metabolic waste from avascular tissues in the eye. Proper homeostasis of AH is required to maintain adequate intraocular pressure as well as optical and refractive properties of the eye. Application of metabolomics to study human AH may improve knowledge about the molecular mechanisms of eye diseases. Until now, global analysis of metabolites in AH has been mainly performed using NMR. Among the analytical platforms used in metabolomics, LC-MS allows for the highest metabolome coverage. The aim of this study was to develop a method for extraction and analysis of AH metabolites by LC-QTOF-MS. Different protocols for AH preparation were tested. The best results were obtained when one volume of AH was mixed with one volume of methanol : ethanol (1 : 1). In the final method, 2 *µ*L of extracted sample was analyzed by LC-QTOF-MS. The method allowed for reproducible measurement of over 1000 metabolic features. Almost 250 metabolites were identified in AH and assigned to 47 metabolic pathways. This method is suitable to study the potential role of amino acids, lipids, oxidative stress, or microbial metabolites in development of ocular diseases.

## 1. Introduction

Metabolomics aims to identify and (semi-)quantify the small molecule metabolites present in a studied sample [1]. This approach has been extensively applied in biomedical research to find disease biomarkers [1], study therapeutic effects of drugs or natural substances with potential therapeutic capabilities [2], or explore metabolic pathways perturbed by a particular disease or condition [3]. Regarding the samples of mammalian origin, blood (serum/plasma) and urine are most commonly sampled for metabolomics. These types of biological fluids are especially important in the field of biomarkers discovery [1]. However, in studies aiming to explore changes in metabolic pathways evoked by the development of a disease, more specific biospecimens could be more informative. Such specific samples may provide more detailed information about disease pathogenesis and progression, toxicity, novel therapeutic targets, and response to drug administration, or about the effects of nutrition and exercise [4]. Just in the case of cancer has metabolomics been applied to study lung, kidney, prostate, gastric, colorectal, ovarian [5], liver, brain [6], and breast cancer tissues [7]. Among other types of mammalian samples metabolomics studies on cerebrospinal [8] or amniotic [9] fluids, exhaled breath condensate [10], stool, or saliva [11] samples were performed.

One of the human fluids in which the composition of small molecules has not been extensively explored using a metabolomics approach is aqueous humor (AH). AH is a transparent fluid produced by the ciliary body that passes from the posterior chamber through the pupil into the anterior chamber, where it drains out of the eye [12]. It supplies nutrients and removes metabolic waste from avascular tissues in the eye [13]. Homeostasis of AH is required to maintain adequate intraocular pressure as well as optical and refractive properties of the eye [14]. AH is a complex mixture known to contain electrolytes, glucose, urea, antioxidants (e.g., glutathione and ascorbic acid), organic solutes, several proteins (e.g., growth factors and cytokines), and oxygen and carbon dioxide [12, 14]. Until now, only a few articles in which metabolomics has been applied to analyze AH have been published [12, 15–18]. Most of those studies were performed on animal models and with the use of nuclear magnetic resonance spectroscopy (NMR) [15–18]. The group of Song et al. have studied the metabolic changes in rabbit AH after glucocorticosteroids administration [15] and the hypotensive effects of glycyrrhizin on a rabbit model of ocular hypertension induced by triamcinolone acetonide [16]. ^1^H NMR has also been used to study metabolic alterations in AH in the rat glaucoma model induced by intracameral sodium hyaluronate injections [17] and to evaluate the effect of UV-A and UV-B irradiation on the metabolic profile of aqueous humor in rabbits [18]. Currently there is only one metabolomics study on human aqueous humor. In this research, a mass spectrometry (MS) based metabolomics approach was used to explore changes in metabolites observed in patients with different severities of myopia [12]. Metabolomics has a huge potential for studying human AH. It may improve knowledge about the molecular mechanisms of several eye diseases (e.g., cataract, glaucoma, pseudoexfoliation syndrome, or age-related macular degeneration) and even indicate metabolic pathways which can be promising drug targets. Moreover, like tissue metabolomics, sampling AH offers benefits of specificity over other biofluids to enable better, more sensitive characterization at the site of the disease.

MS and NMR are the analytical platforms that dominate within the field of metabolomics. The concept of global analysis of small molecules was first proposed in 1999 by Nicholson et al. who used NMR for this purpose [19]. NMR is a highly selective, nondestructive technique; however it offers lower sensitivity in comparison to MS. On the other hand, detection of metabolites using MS is usually preceded by their separation using liquid chromatography (LC), gas chromatography (GC), or capillary electrophoresis (CE). All three separation techniques in combination with MS detection are used in metabolomics studies [20]. GC-MS is applied to measure volatile and thermally stable analytes and CE-MS is applied for polar and charged molecules, while LC-MS is applied for polar and nonpolar compounds (depending on the type of column used) [21]. Separation step reduces the complexity of the biological sample and allows the analysis of different classes of metabolites at different time points [22]. Among the analytical platforms utilized in metabolomics, LC-MS allows for the highest metabolome coverage [1]. The first technique allowed for detection of about 20 metabolites in AH [16], while, using CE-MS and LC-MS, tens and hundreds of metabolites were detected, respectively [12]. The number of metabolites detected by LC-MS depends on the sample extraction procedure and chromatographic conditions used [23].

The aim of this study was to develop a strategy for the extraction of metabolites from AH and their analysis by LC-QTOF-MS which allows for detection of the highest number of metabolites by a single analytical platform. Considering similarity between AH and plasma [14] reversed phase chromatography was selected.

## 2. Materials and Methods

### 2.1. Samples

AH samples were collected from the patients undergoing cataract surgery. The study was approved by the Medical Ethics Committee of the Medical University of Bialystok and conformed to the tenets of the Declaration of Helsinki. Collection of the samples was performed following participants' written informed consent for participation in the study. The anterior chamber of the eye was punctured using a 30 G needle and approximately 100–200 *μ*L of AH was aspirated, transferred to Eppendorf® tubes (Eppendorf, Hamburg, Germany), frozen, and stored at −80°C until the day of analysis.

### 2.2. Chemicals and Reagents

Purified water was obtained using the Milli-Q Integral 3 system (Millipore SAS, Molsheim, France). LC-MS grade methanol, acetonitrile, formic acid and LC grade acetone, and ethanol were purchased from Sigma-Aldrich Chemie GmbH, Steinheim, Germany. The API-TOF reference mass solution kit (G1969-850001) and tuning solutions, ESI-L low concentration tuning mix (G1969-85000), and ESI-TOF Biopolymer Analysis reference masses (G1969-850003) were purchased from Agilent Technologies, Santa Clara, California, USA.

### 2.3. Sample Treatment and Analysis

On the day of analysis, AH samples were defrosted on ice. Protein precipitation and metabolite extraction were performed by vortex-mixing (IKA-Werke GmbH & Co. KG, Staufen, Germany) (1 min) one volume of the defrosted sample with the addition of one volume of freeze cold (−20°C) methanol/ethanol (1 : 1) mixture. After extraction, samples were stored on ice for ten minutes and centrifuged (Eppendorf, Hamburg, Germany) at 21,000 ×g for 10 minutes at 4°C. The supernatant was filtered through a 0.22 *μ*m nylon filter (ThermoFisher Scientific, Waltham, Massachusetts, USA). Blank extraction was also prepared and analyzed together with AH samples. Blank extraction was analyzed in triplicate, while analysis of AH sample was five times repeated.

Samples were analyzed by an LC-MS system consisting of 1290 Infinity LC (Agilent, Santa Clara, California, USA) with a degasser, two binary pumps, and a thermostated autosampler coupled to a 6550 Q-TOF-MS detector (Agilent, Santa Clara, California, USA). Analyses were performed in ESI+ and ESI− modes, whereby 2 *μ*L of extracted AH samples was injected into a thermostated (30°C) RP Poroshell 120 EC-C18, 3.0 × 100 mm, 2.7 *μ*m column (Agilent Technologies, Santa Clara, California, USA). The flow rate was 0.5 mL/min with solvent A (water with 0.1% formic acid) and solvent B (acetonitrile with 0.1% formic acid). The gradient started at 1% phase B for the first minute, followed by an increase of phase B to reach 100% in 10 min. After reaching 100% the gradient returned to starting conditions (1% of phase B) in 0.1 min and was maintained at this solvents proportion for 4.9 min in order to reequilibrate the system for the next injection. During the method development longer gradients (12.5, 15, and 20 min) were also tested.

The mass spectrometer was operated in full scan mode from* m/z* 50–1000. The capillary voltage was set to 3 kV; the drying gas flow rate was 12 L/min at 250°C and gas nebulizer at 45 psig; fragmentor voltage was 225 V for positive and 275 V for negative ionization mode. Data was collected in centroid mode at a scan rate of 2 spectra per second. Accurate mass measurements were obtained by means of calibrant solution delivery using a dual-nebulizer ESI source. A calibrating solution (G1969-85000) containing reference masses at* m/z* 121.0509 (protonated purine) and* m/z* 922.0098 (protonated hexakis (1H,1H,3H-tetrafluoropropoxy)phosphazine or HP-921) in positive ion mode or* m/z* 119.0363 (proton abstracted purine) and* m/z* 966.0007 (formate adduct of HP-921) in negative ion mode was continuously introduced by an isocratic pump (Agilent, Santa Clara, California, USA) at a flow rate of 0.5 mL/min (1 : 100 split).

### 2.4. Data Treatment

The raw data collected by the analytical instrumentation was cleaned of background noise and unrelated ions by the Molecular Feature Extraction (MFE) tool in Mass Hunter Qualitative Analysis Software (B.06.00, Agilent, Santa Clara, California, USA). The MFE algorithm uses the accuracy of the mass measurements to group ions related by charge-state envelope, isotopic distribution, and/or the presence of adducts and dimers. The MFE then creates a list of all possible components as represented by the full TOF mass spectral data. Each compound is described by mass, retention time (RT), and abundance. The limit for the background noise was set to 1000 counts for data extraction by MFE and the following adduct settings were applied to identify coeluting adducts of the same feature: +H, +Na, +K in positive ion mode and −H, +HCOO, +Cl for negative ion mode. Dehydration neutral losses were also allowed in both ionization modes. The sample alignment and data filtering were performed using Mass Profiler Professional 12.6.1 (Agilent, Santa Clara, California, USA). Parameters applied for the alignment were 1% for RT and 10 ppm for the mass variation. Data obtained in every step of the study were filtered to keep metabolic features present in every analysis of blank extract and in 100% of the last three analyses of extracted AH sample.

### 2.5. Metabolite Identification

Identification of compounds detected by LC-MS was performed as follows. Accurate masses of features were searched against the METLIN, KEGG, LIPIDMAPS, and HMDB databases, which were simultaneously accessed by CEU Mass Mediator (available on-line search engine, http://ceumass.eps.uspceu.es/mediator/). Putative identities were then confirmed by LC-MS/MS using a QTOF (Agilent, Santa Clara, California, USA). Ions corresponding to putatively identified metabolites were targeted for collision-induced dissociation (CID) fragmentation based on the previously determined exact mass and RT. Primary identification was achieved by matching accurate mass and isotopic distribution of the targeted ion. In the second step of identification MS/MS fragmentation spectra were studied to elucidate the structure of the fragmented molecules or were compared with the spectral data of reference compounds available at public databases (HMDB, METLIN, and LIPIDMAPS).

## 3. Results and Discussion

### 3.1. Optimization of the Experimental Conditions

**Figure 1:**
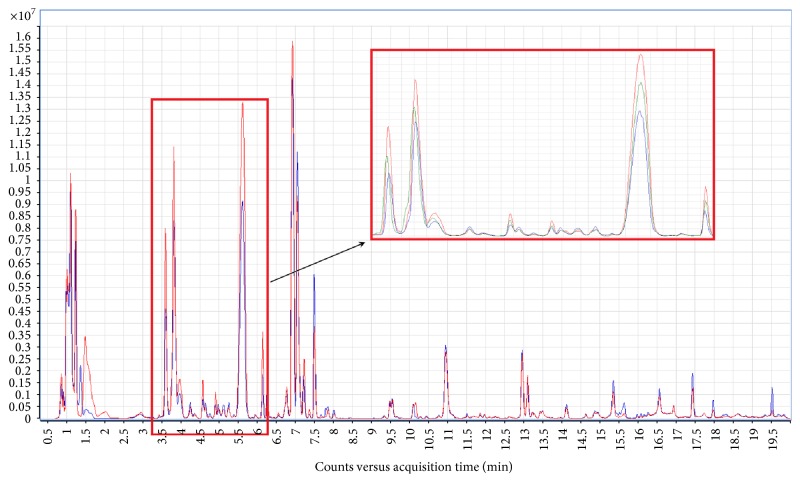
Total compound chromatograms obtained for AH samples extracted with different volumes of methanol : ethanol. Aqueous humor samples were prepared by addition of one AH volume to one (red), two (green), or three (blue) volumes of methanol : ethanol and analyzed with 20 min gradient.

The purpose of this study was to develop a method for metabolic fingerprinting of human AH. LC-MS was selected as the analytical platform which allows measurement of the largest number of metabolites. However, during the method development not only the number of metabolites detected but also the reproducibility of measured intensities of metabolic features and the number of features in the blank extractions were taken into account. AH is to some extent similar to blood plasma as it contains plasma proteins and metabolites which are filtered to AH through fenestrated capillaries of the ciliary body stroma* via* the iris root [14]. Although the total concentration of proteins in AH seems to be low (five times lower than in blood plasma [14]), protein depletion in AH sample was performed before metabolite analysis. This step was used to eliminate potential enzymatic reactions between proteins and metabolites. The lower complexity of the sample is also advantageous as it minimizes matrix effect compared to the analysis of blood plasma, which may affect chromatographic resolution and ionization of metabolites in the ion source [29]. Moreover, injection of the sample containing proteins into LC system with acetonitrile or alcohol as the organic mobile phase may lead to system clogging by precipitated proteins [30]. Consequently, in the first step, different solvents and their mixtures commonly used for simultaneous proteins precipitation and metabolites extraction [31] were tested. One volume of AH was mixed with one volume of the following solutions: acetone, acetonitrile, methanol : ethanol (1 : 1), and methanol/acetone/acetonitrile (1 : 1 : 1). Extractions were performed as described in Materials and Methods. AH extracts and blank extraction samples were analyzed with a 20 min gradient in both ion modes. As it can be seen in Table 1 the numbers of metabolic features detected in each extract were similar. The highest number was detected in the acetone extract (1200 metabolic features), while the lowest was detected in acetonitrile extract (1102 metabolic features). Reproducibility of the method was also evaluated by calculation of coefficient of variation (CV) for intensity of each detected feature. The results were presented as the percentage of the features with CV ≤ 20% or CV ≤ 30%. The most reproducible results (92.8% of metabolic features with coefficient of variation (CV) < 30% and 87.9% with CV < 20%) were obtained with methanol : ethanol extraction. Moreover, this extraction allowed for detection of 1165 metabolic features and was selected as appropriate for AH preparation. In the next step different volumes of methanol : ethanol for protein precipitation and metabolite extraction were tested. Samples were prepared by mixing 1 volume of AH with 0.5, 1, 2, or 3 volumes of methanol : ethanol and analyzed with a 20 min gradient. The lowest number (about one thousand) of metabolic features was detected in the samples prepared in the ratio of AH to methanol : ethanol equal to 2 : 1. The other samples (ratios 1 : 1, 1 : 2, and 1 : 3) gave similar results (from 1074 to 1184 metabolic features); however, the highest reproducibility (94.1% of metabolic features with CV < 30% and 89.8% with CV < 20%) was obtained with 1 : 1 AH to solvent ratio. Moreover, as can be seen in Figure 1, chromatograms recorded for samples prepared in the ratio 1 : 1 were the most intense, which can be crucial for identification of metabolites by MS/MS fragmentation. Consequently, mixing one volume of sample with one volume of methanol : ethanol was selected as an adequate ration for preparation of AH for LC-MS-based metabolic fingerprinting. To decrease the time and consequently the costs of analyses, attempts to shorten the time of analysis were undertaken. The slope of the gradient was increased and different methods in which phase B reached 100% in 10, 12.5, or 15 min were tested. As can be seen in Table 2, the number of metabolic features detected and their repeatability do not differ significantly between the tested methods. The use of a shorter method allows for analysis of larger number of the samples in one sequence, if necessary, offering the potential of this method as a high-throughput screening approach for ocular diseases.

### 3.2. Identification of Metabolites

**Figure 2:**
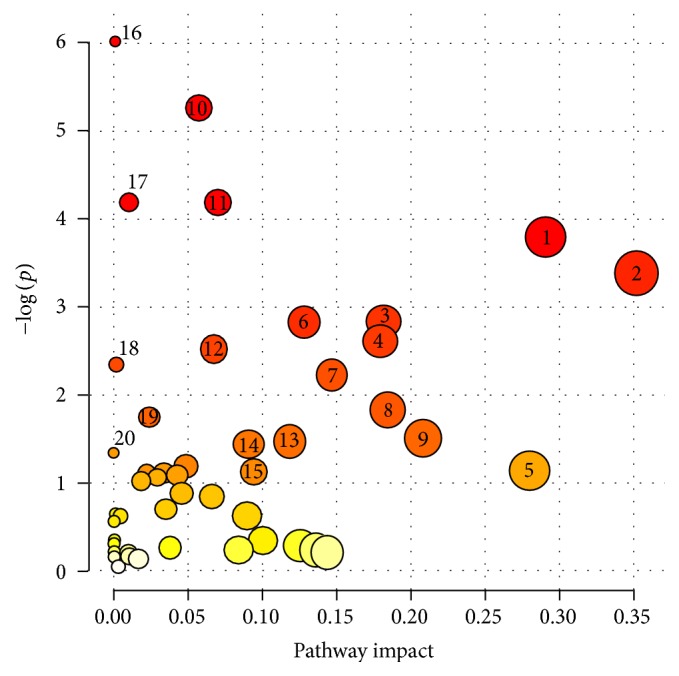
Metabolic pathway analysis. Metabolic pathway analysis was performed with MetaboAnalyst 3.0. Calculated* p* value was established based on the pathway enrichment analysis while pathway impact value based on the pathway topology analysis. Twenty of the most significant pathways are marked with the numbers: 1. phenylalanine metabolism, 2. taurine and hypotaurine metabolism, 3. arginine and proline metabolism, 4. pantothenate and CoA biosynthesis, 5. pyruvate metabolism, 6. biotin metabolism, 7. glyoxylate and dicarboxylate metabolism, 8. tryptophan metabolism, 9. alanine, aspartate, and glutamate metabolism, 10. aminoacyl-tRNA biosynthesis, 11. valine, leucine, and isoleucine metabolism, 12. beta-alanine metabolism, 13. ascorbate and aldarate metabolism, 14. sphingolipid metabolism, 15. glycolysis and gluconeogenesis, 16. nitrogen metabolism, 17. phenylalanine, tyrosine, and tryptophan biosynthesis, 18. glycine, serine, and threonine metabolism, 19. pyrimidine metabolism, and 20. arginine and ornithine metabolism.

The final method for AH fingerprinting with LC-MS allowed for detection of more than one thousand metabolic features with good repeatability. To prove that detected metabolites are not artifacts, metabolic features measured with CV < 20% in three AH analyses were searched against available internet databases. The list of putatively identified features included almost 250 different metabolites. Compounds identified as drugs (or drug metabolites) which were not taken by the patients in addition to phytometabolites were not included in that list. To give an overview of the metabolic pathways to which putatively identified metabolites belong, a pathway analysis was performed using MetaboAnalyst 3.0 (Figure 2 and Table 3). In total, these metabolites could be assigned to 47 metabolic pathways. Based on the pathway enrichment analysis (*p values*) and pathway topology analysis (pathway impact values) the importance of each metabolic pathway can be established [32]. As can be seen in Figure 2, based on metabolites detected in AH, phenylalanine metabolism and taurine and hypotaurine metabolism are the most significant metabolic pathways observed using this method. The value of other pathways is also represented on Figure 2, where 20 of the most significant pathways are marked. The complete list of metabolic pathways to which putatively identified metabolites from AH belong is presented in Table 3. The table includes the number of metabolites present in the pathway and detected in AH as well as results of pathway analysis (*p value* and pathway impact value).

Identification of metabolites forwarded for pathway analysis was putative that, according to the Metabolomics Standards Initiative (MSI), is the lowest level of metabolite identification [33]. To increase the confidence of identification to level 2 [33], MS/MS fragmentation spectra of putatively identified signals were acquired. Obtained fragmentation spectra were compared to spectra available in databases (METLIN, HMDB) or in the literature [3, 34, 35]. Based on that information, the identity of over fifty metabolites was confirmed. Identified compounds are summarized in Tables 4–6 including retention time, theoretical mass and error of measured mass, MS/MS fragments, average intensity, and calculated coefficient of variation. Except metabolites four pharmacological substances (Table 4) present in eye drops or eye gels, which were administrated to the patients, were also detected in AH. Identified metabolites could be grouped into lipid compounds (fatty acids, acylcarnitines, sphingolipids, and fatty acid amides) and amino acids which are included in Table 5. The rest of the identified metabolites are summarized in Table 6.

### 3.3. Relationship between Identified Metabolites and Ocular Abnormalities

Considering the number of metabolic pathways to which detected metabolites belong (Table 3 and Figure 2) and diversity of the identified metabolites (Tables 4–6), the proposed method for AH fingerprinting could be useful to study several eye diseases. Amino acids and lipids have already been linked with myopia [12], glaucoma [17], or ocular hypertension [16]. Proteomics studies on AH showed that APOC1 (one of apolipoproteins, proteins involved in lipid transport and metabolism) was overexpressed in patients with Coats' disease [36], while another apolipoprotein (APOD) was found decreased in AH of patients with pseudoexfoliation syndrome [14]. Consequently, our method for AH fingerprinting could also be used to study metabolic changes in AH related to these diseases providing additional information about lipid metabolism in Coats' disease and pseudoexfoliation syndrome. Among other metabolites detected in AH (Table 6) several molecules with antioxidative properties (e.g., citric acid, ascorbic acid, quinic acid, pantothenic acid, betaine, or taurine) or related to oxidative stress (e.g., pyroglutamic acid or hypoxanthine) were identified. Oxidative stress contributes to several eye diseases, which have been reviewed recently [37]. The knowledge about the role of oxidative stress in development and progression of these diseases could also be extended by application of this method. Some of the identified metabolites (e.g., p-cresol, p-cresol sulfate, or indoxylsulfuric acid) are known to be linked with gut microbiota [25, 26]. Currently there is only one scientific report indicating the contribution of gut bacteria to eye disease. Horai et al. demonstrated in a mouse model of spontaneous uveitis that a microbiota-dependent signal activates retina-specific T cells in the gut lamina propria that precedes clinical onset of the autoimmune uveitis, important cause of visual impairment in humans [38]. Metabolomics of AH may also help to evaluate a possible role of microbial metabolites in pathogenesis of eye diseases. The list of identified metabolites includes also such molecules which have already been reported as altered in different eye disorders. Changes in choline, acetylcholine, arginine, and phenylalanine levels were observed in tears of humans with dry eye disorders (DEDs) in comparison to control group [24], while alterations in lactate have been found in uveitis [27] or ocular hypertension [28].

## 4. Conclusions

Currently in only one study metabolic fingerprinting with LC-QTOF-MS was used for global measurement of metabolites in AH. In this report AH was 5 times diluted with water and 20 *μ*L of the sample was analyzed by LC-QTOF-MS with reversed phase (RP) chromatographic separation [12]. In the present study for the first time several protocols for AH sample preparation before metabolic fingerprinting analysis were tested. Different solvents for simultaneous protein precipitation and metabolites extraction from AH were used. The best results were obtained with a mixture of methanol : ethanol (1 : 1). In the final method AH sample was two times diluted and 2 *μ*L of extracted sample was analyzed using RP chromatography with QTOF-MS detection. Using the method, over 1000 metabolic features were reproducibly measured from which almost 250 were identified putatively and the identity of over 50 was confirmed by MS/MS fragmentation. Identified metabolites were assigned to 47 metabolic pathways. This method revealed the extent of the potential role of amino acids, lipids, oxidative stress, or microbial metabolites in development of eye diseases.

## Figures and Tables

**Table 1:** The number of metabolic features detected in different aqueous humor extracts.

Extraction solutions	Number of metabolic features detected	The % of metabolic features measured with given reproducibility	
		CV < 30%	CV < 20%
Acetonitrile	1102	92.4%	87.6%
Acetone	1200	91.2%	85.4%
Methanol/acetone/acetonitrile	1196	91.0%	85.9%
Methanol/ethanol	1165	92.8%	87.9%

In mixed solutions the solvents were used in equal proportions. The numbers of metabolic features detected do not include features present in the blank extraction. Presented results include data from both ion modes.

**Table 2:** The number of metabolic features detected with different slopes of the gradient.

Method	Number of metabolic features detected			The % of reproducibly measured metabolic features	
	Total	CV < 30%	CV < 20%	CV < 30%	CV < 20%
15 min	1445	1346	1283	93.1%	88.8%
17.5 min	1311	1226	1188	93.5%	90.6%
20 min	1407	1319	1300	93.7%	92.4%

The numbers of metabolic features detected do not include features present in the blank extraction. Presented results include data from both ion modes.

**Table 3:** The list of metabolic pathways to which putatively identified metabolites belong in aqueous humor.

Pathway	Number of metabolites in pathway	Number of metabolites detected in AH	*p* value	Pathway impact
Nitrogen metabolism	39	6	0.034324	0
Aminoacyl-tRNA biosynthesis	75	8	0.022482	0.05634
Phenylalanine, tyrosine, and tryptophan biosynthesis	27	4	0.32793	0.008
Valine, leucine, and isoleucine biosynthesis	27	4	0.21809	0.06784
Phenylalanine metabolism	45	5	0.15979	0.28993
Taurine and hypotaurine metabolism	20	3	0.059751	0.35252
Biotin metabolism	11	2	0.073415	0.13008
Arginine and proline metabolism	77	6	0.10756	0.18287
Pantothenate and CoA biosynthesis	27	3	0.82929	0.18014
Beta-alanine metabolism	28	3	0.80869	0.06625
Glycine, serine, and threonine metabolism	48	4	0.058661	0.00118
Glyoxylate and dicarboxylate metabolism	50	4	0.76878	0.14716
Tryptophan metabolism	79	5	0.22562	0.18481
Pyrimidine metabolism	60	4	0.69873	0.02251
Alanine, aspartate, and glutamate metabolism	24	2	0.31427	0.20703
Ascorbate and aldarate metabolism	45	3	0.23176	0.11744
Sphingolipid metabolism	25	2	0.52665	0.09061
D-Arginine and D-ornithine metabolism	8	1	0.77737	0
Tyrosine metabolism	76	4	0.01538	0.04724
Glycolysis or gluconeogenesis	31	2	0.080131	0.09576
Pentose phosphate pathway	32	2	0.43366	0.02181
Vitamin B6 metabolism	32	2	0.005252	0.0303
Glycerolipid metabolism	32	2	0.30138	0.0412
Pyruvate metabolism	32	2	0.40718	0.28201
D-Glutamine and D-glutamate metabolism	11	1	0.32793	0.02674
Methane metabolism	34	2	0.75986	0.01696
Glycosylphosphatidylinositol- (GPI-) anchor biosynthesis	14	1	0.48974	0.0439
Valine, leucine, and isoleucine degradation	40	2	0.32793	0.06442
Sulfur metabolism	18	1	0.33673	0.03307
Citrate cycle (TCA cycle)	20	1	0.17427	0.09024
Caffeine metabolism	21	1	0.32793	0
Selenoamino acid metabolism	22	1	0.35501	0.00321
Thiamine metabolism	24	1	0.87881	0
Lysine biosynthesis	32	1	0.85927	0.09993
Terpenoid backbone biosynthesis	33	1	0.82929	0
Ubiquinone and other terpenoid-quinone biosynthesis	36	1	0.01538	0
Glutathione metabolism	38	1	0.56091	0.03608
Glycerophospholipid metabolism	39	1	0.96454	0.12753
Butanoate metabolism	40	1	0.095918	0.08516
Nicotinate and nicotinamide metabolism	44	1	0.002484	0
Histidine metabolism	44	1	0.258	0.13988
Primary bile acid biosynthesis	47	1	0.5441	0.00822
Lysine degradation	47	1	0.59272	0.14675
Purine metabolism	92	2	0.70989	0.00969
Pentose and glucuronate interconversions	53	1	0.74098	0
Cysteine and methionine metabolism	56	1	0.80869	0.01649
Amino sugar and nucleotide sugar metabolism	88	1	0.86413	0.00265

Pathway impact and *p* value were obtained from metabolic pathway analysis performed with MetaboAnalyst 3.0.

**Table 4:** The list of drugs identified in aqueous humor.

Compound	RT [min]	Monoisotopic mass [Da]	Mass error [ppm]	Fragments	Abundance [counts]	CV [%]
Dexpanthenol	3.1	205.1314	5	P: 188.128, 170.117, 102.054, 76.075, 74.023, 69.069, 58.064	4.1*E* + 07	0
	3.1	205.1314	1	N: 174.114, 156.103, 126.092, 102.056, 74.061, 72.046, 71.05, 44.014	1.6*E* + 07	1
Timolol	4.4	316.1569	2	P: 261.102, 244.076, 188.048, 74.06, 56.049	9.1*E* + 06	0
Tropicamide	4.1	284.1525	3	P: 267.149, 255.149, 135.092	1.4*E* + 08	1
Proparacaine	4.8	294.1943	3	P: 222.113, 178.087, 136.039, 100.112	6.5*E* + 07	1

P or N indicates polarity in which metabolite was detected, positive or negative, respectively. Abundance is a value representing metabolite intensity calculated by MFE algorithm.

**Table 5:** The list of lipids and amino acids identified in aqueous humor.

Compound	RT [min]	Monoisotopic mass [Da]	Mass error [ppm]	Fragments	Abundance [counts]	CV [%]	Metabolic pathway or relation to eye diseases
Acetylcarnitine	1.2	203.1158	6	P: 145.049, 85.028, 60.08	1.2*E* + 07	7	FAO
Butyrylcarnitine	3.4	231.1471	4	P: 173.08, 85.028, 60.08	1.6*E* + 06	4	FAO
Hexanoylcarnitine	4.6	259.1784	2	P: 99.082, 85.027	8.7*E* + 04	2	FAO
Octanoylcarnitine	5.6	287.2097	5	P: 229.141, 127.11, 85.029	1.4*E* + 05	1	FAO
Decanoylcarnitine	6.4	315.241	1	P: 85.028, 60.081	5.8*E* + 04	3	FAO
Palmitic amide	6.7	255.2562	2	P: 102.089, 88.075, 57.069, 43.052	8.5*E* + 04	1	Endocannabinoids metabolism
Stearamide	7.6	283.2875	1	P: 88.076	3.1*E* + 04	15	Endocannabinoids metabolism
Hexadecasphinganine	6.6	273.2668	4	P: 256.263, 106.086, 88.075, 57.069	1.6*E* + 07	4	Sphingolipids metabolism
Hydroxysphinganine	6.7	317.293	1	P: 300.29, 256.264, 88.075, 57.069	7.1*E* + 04	15	Sphingolipids metabolism
5 : 1 dicarboxylic fatty acid	1.2	130.0266	2	N: 85.03, 41.039	3.4*E* + 05	17	Fatty acid metabolism
Suberic acid	4.6	174.0892	3	N: 111.08, 83.051	5.6*E* + 04	6	Fatty acid metabolism
Nonanedioic acid	5.1	188.1049	3	N: 169.091, 125.097, 97.064	1.6*E* + 05	5	Fatty acid metabolism
Sebacic acid	5.6	202.1205	3	N: 183.101, 139.112	3.9*E* + 04	4	Fatty acid metabolism
Undecanedioic acid	6.1	216.1362	3	N: 197.12, 153.13	3.6*E* + 04	3	Fatty acid metabolism
Undecanedicarboxylic acid	7.0	244.1675	2	N: 225.15, 181.16	4.5*E* + 04	1	Fatty acid metabolism
Arginine	1.0	174.117	5	P: 158.093, 130.097, 116.071, 70.065, 60.055	1.9*E* + 06	1	Arginine and proline metabolism, urea cycle, Canavan disease, DEDs [24]
Glutamine	1.0	146.0691	4	P: 130.049, 101.07, 102.054, 84.044, 56.049, 41.038	3.2*E* + 04	19	Nitrogen, arginine, proline, pyrimidine, alanine, aspartate, glutamate, glutamine and purine metabolism, aminoacyl-tRNA biosynthesis
	1.0	146.0691	1	N: 128.036, 127.051, 109.041, 101.071, 99.056, 84.046, 74.025, 58.03, 41.998	5.0*E* + 05	5	
Histidine	1.0	155.0695	5	P: 110.072, 93.044, 83.06	1.6*E* + 05	1	Nitrogen, alanine, and histidine metabolism, aminoacyl-tRNA biosynthesis
Phenylalanine	3.0	165.079	4	P: 149.059, 131.048, 120.08, 103.053	2.1*E* + 07	0	Nitrogen and phenylalanine metabolism; aminoacyl-tRNA, phenylalanine, tyrosine, and tryptophan biosynthesis; DEDs [24]
	3.0	165.079	7	N: 147.045, 103.055, 72.009	1.6*E* + 06	2	
Tryptophan	3.6	204.0899	0	N: 186.057, 159.093, 142.067, 116.05, 74.024	1.2*E* + 06	1	Nitrogen, tryptophan, glycine, serine, and threonine metabolism; aminoacyl-tRNA, phenylalanine, tyrosine, and tryptophan biosynthesis

P or N indicates polarity in which metabolite was detected, positive or negative, respectively. Abundance is a value representing metabolite intensity calculated by MFE algorithm. FAO: fatty acid oxidation; DEDs: dry eye disorders.

**Table 6:** The list of other metabolites detected in aqueous humor.

Compound	RT [min]	Monoisotopic mass [Da]	Mass error [ppm]	Fragments	Abundance [counts]	CV [%]	Metabolic pathway or relation to eye diseases
Spermidine	0.8	145.1579	4	P: 129.14, 112.111, 84.035, 75.091, 72.081, 58.064	8.2*E* + 04	1	Alanine metabolism
Hydroxyglutaric acid	1.2	148.0372	4	N: 129.021, 103.04, 101.024, 85.031, 57.035	4.0*E* + 05	6	Butanoate metabolism
Aconitic acid	1.2	174.0164	0	N: 129.021, 111.01, 85.03, 41.04	1.1*E* + 05	14	Glyoxylate and dicarboxylate metabolism
Citric acid	1.2	192.027	1	N: 129.02, 111.01, 87.01, 85.031	4.4*E* + 04	5	Glyoxylate and dicarboxylate metabolism, TCA
Ascorbic acid	1.2	176.0321	1	N: 127.004, 115.004, 87.009, 71.014, 59.014	2.1*E* + 07	5	Glutathione, ascorbate and aldarate metabolism
Pyroglutamic acid	1.2	129.0426	3	N: 82.028	1.3*E* + 05	1	Glutathione metabolism
Indoleacrylic acid	3.6	187.0633	6	P: 170.06, 146.06, 142.065, 15.054	1.1*E* + 06	7	Microbial metabolism [25, 26]
Hydroxyphenyllactic acid	3.7	182.0579	3	N: 163.04, 135.045, 119.05, 107.049, 92.99, 72.99, 44.996	2.0*E* + 05	2	Tyrosine metabolism
Phenylacetylglutamine	4.1	264.111	0	N: 145.062, 127.052	5.7*E* + 05	0	Phenylalanine metabolism
Trimethylamine N-oxide	1.0	75.0684	6	P: 58.065, 42.033, 30.032	1.0*E* + 06	1	Methane metabolism
p-Cresol	4.6	108.0575	4	N: 106.042, 105.033, 92.028, 77.038	1.4*E* + 05	2	Degradation of aromatic compounds
p-Cresol sulfate	4.65	188.0143	1	N: 107.05, 79.957	1.4*E* + 06	2	Degradation of aromatic compounds
Uric acid	1.2	168.0283	3	P: 151.094, 141.04	7.7*E* + 05	6	Purine metabolism
	1.2	168.0283	5	N: 124.016, 96.021, 69.01, 41.999	2.5*E* + 06	0	
Aminosalicylic acid or hydroxyanthranilic acid	5.2	153.0426	6	P: 136.039, 108.044, 80.05	1.0*E* + 07	0	Tryptophan metabolism
Quinic acid	1.1	192.0634	0	N: 173.046, 127.041, 93.035, 85.03, 59.014, 44.997	1.8*E* + 05	1	Phenylalanine, tyrosine, and tryptophan biosynthesis
Choline	1.0	103.0997	2	P: 60.081, 45.033	9.2*E* + 04	2	Phospholipid biosynthesis, DEDs [24]
Creatine	1.1	131.0695	7	P: 90.055	2.1*E* + 07	0	Arginine and proline metabolism
Betaine	1.0	117.079	6	P: 59.073, 58.065	7.9*E* + 06	2	Methionine metabolism
Indole	3.6	117.0578	5	P: 117.057, 100.112, 91.054	1.8*E* + 06	0	Tryptophan metabolism; phenylalanine, tyrosine, and tryptophan biosynthesis
Pantothenic acid	3.2	219.1107	2	N: 146.081, 99.045, 88.04, 71.05, 44.014	2.3*E* + 05	19	Alanine metabolism; pantothenate and CoA biosynthesis
Hydroxybenzaldehyde	4.5	122.0368	3	N: 120.022, 92.027	3.5*E* + 05	1	Degradation of aromatic compounds
Glycolic acid	1.0	76.016	3	N: 72.994, 47.013	2.4*E* + 06	7	Glycine, serine, threonine, glyoxylate, and dicarboxylate metabolism; pentose phosphate pathway
Threonic acid	1.0	136.0372	0	N: 117.02, 89.025, 75.009, 59.014	2.1*E* + 07	1	Ascorbate and aldarate metabolism
Hydroxypyruvic acid	1.0	104.011	3	N: 59.014, 41.002, 31.019	2.0*E* + 05	0	Glycine, serine, threonine, glyoxylate, and dicarboxylate metabolism
Glyceric acid	1.1	106.0266	2	N: 75.009, 59.013, 56.998	1.2*E* + 06	3	Glycerolipid, glyoxylate, and dicarboxylate metabolism; pentose and glucuronate interconversion
Carnitine	1.0	161.1052	5	P: 103.039, 102.091, 85.028, 60.081, 43.017	1.3*E* + 07	1	Glycine, serine, and threonine metabolism
Trigonellinamide	1.0	136.0637	4	P: 94.064, 92.049	8.7*E* + 04	1	Nicotinate and nicotinamide metabolism
Pyrrolidonecarboxylic acid	1.0	129.0426	1	P: 84.044, 56.049, 41.038	1.0*E* + 05	3	Glutathione metabolism
Taurine	1.0	125.0147	0	N: 79.958	1.7*E* + 05	3	Nitrogen, taurine, and hypotaurine metabolism; primary bile acid biosynthesis
Acetylcholine	1.1	145.1103	7	P: 87.0437, 60.08, 43.017	8.0*E* + 05	1	Phospholipid biosynthesis, DEDs [24]
Dimethylarginine	1.1	202.1430	5	P: 158.129, 116.07, 88.086, 70.065, 46.064	5.6*E* + 05	0	Protein methylation process
Acetyl-histidine	1.1	197.0800	4	P: 180.081, 156.076, 152.081, 110.071,	2.5*E* + 05	1	Nitrogen metabolism
Hypoxanthine	1.2	136.0385	2	P: 119.035, 110.034, 94.039, 92.057, 82.039, 55.028	3.2*E* + 05	3	Purine metabolism
Lactic acid	1.2	90.0317	1	N: 43.019	1.8*E* + 05	13	Pyruvate metabolism, uveitis [27], ocular hypertension [28]
Glutamylleucine	3.6	260.1372	2	N: 130.089	5.0*E* + 04	3	Nitrogen metabolism
Indoxylsulfuric acid	4.2	213.0096	3	N: 132.046, 80.964, 79.957	5.4*E* + 04	6	Tryptophan metabolism

P or N indicates polarity in which metabolite was detected, positive or negative, respectively. Abundance is a value representing metabolite intensity calculated by MFE algorithm. TCA: tricarboxylic acid cycle; DEDs: dry eye disorders.
